# Two Agents in the Brain: Motor Control of Unimanual and Bimanual Reaching Movements

**DOI:** 10.1371/journal.pone.0010086

**Published:** 2010-04-08

**Authors:** Tomohisa Asai, Eriko Sugimori, Yoshihiko Tanno

**Affiliations:** Department of Cognitive and Behavioral Science, Graduate School of Arts and Sciences, University of Tokyo, Tokyo, Japan; University of Regensburg, Germany

## Abstract

Previous studies have suggested that the left and right hands have different specialties for motor control that can be represented as two agents in the brain. This study examined how coordinated movements are performed during bimanual reaching tasks to highlight differences in the characteristics of the hands. We examined motor movement accuracy, reaction time, and movement time in right-handed subjects performing a three-dimensional motor control task (visually guided reaching). In the no-visual-feedback condition, right-hand movement had lower accuracy and a shorter reaction time than did left-hand movement, whereas bimanual movement had the longest reaction time, but the best accuracy. This suggests that the two hands have different internal models and specialties: closed-loop control for the right hand and open-loop control for the left hand. Consequently, during bimanual movements, both models might be used, creating better control and planning (or prediction), but requiring more computation time compared to the use of one hand only.

## Introduction

In everyday life, the non-preferred hand appears to play a supportive role to the preferred hand, as we see in the “waiter task” [1] in which a waiter is asked to use the arm of the non-preferred hand to balance a tray while using the preferred hand to carefully lift a selected glass from the tray and place that glass in front of a customer. However, some studies have reported that right-handers perform a number of tasks better with the left hand, e.g., the processing of tactile information [2], [3]. Other studies have indicated that unimanual aiming movements were planned faster and controlled more accurately for the left (or non-preferred) than for the right (or preferred) hand [4]–[9]. Although the performances of preferred and non-preferred hands are often controversial (opposing results have often been seen, see for review [10], [11]), there is no doubt that left and right hands perform differently. Indeed, the system underpinning the right hand may be superior in processing sensory feedback [12], and the left hemisphere may be superior in gauging the forces propelling movements [13]. Nevertheless, according to traditional views on the dichotomy between the hemispheres, the right hemisphere is more involved in spatial activities (e.g., producing smaller movement errors [14]), whereas the left hemisphere is more involved in temporal activities (e.g., more rapid sequential processing [15]) (cf. [10], [16]). In this context, one might hypothesize that reaching with the right hand would be associated with more rapid responses, and reaching with the left hand would be associated with greater accuracy when tasks involve both accuracy and speed simultaneously. However, inconsistent results, depending on experimental circumstances, have emerged. These inconsistencies have included differences among studies in the cognitive–motor requirements of the tasks (e.g. single vs. sequential aiming or target size; cf. [10]).

Although many studies have suggested that each hand has different special talents, to the best of our knowledge, no study, except for those conducted under specific conditions (e.g., a waiter task), has examined how coordinated movements involving both hands are performed. If the two hands have different specialties, some benefit might accrue to bimanual coordination during the same movement, such as reaching for an object using both hands, as if the two agents were helping each other. This study examined bimanual reaching to identify the benefits associated with such coordinated movements, as visually guided reaching may be the most basic action of humans. Why, for example, do baseball batters swing their bats with both hands? Although most studies have examined performance in the single preferred hand [17]–[19], coordinated bimanual actions form the basis for many everyday motor skills. Therefore, we examined for the first time whether bimanual coordination enhances the accuracy of one's own arm movements or affects reaction time and movement time either with or without visual feedback.

## Methods

### Participants, Apparatus, and Stimuli

**Figure 1:**
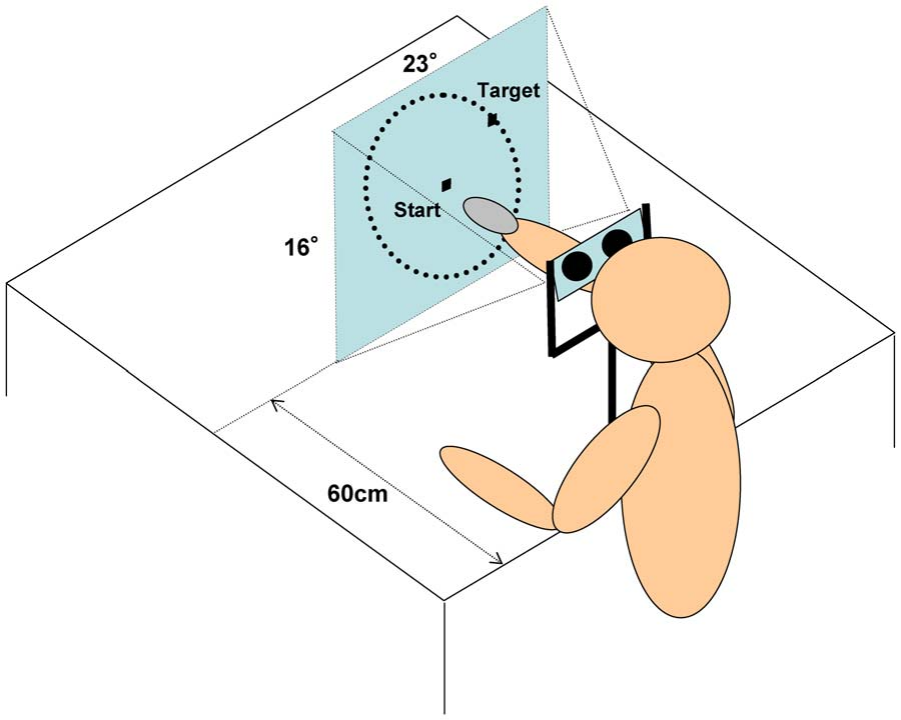
Illustration of the experimental apparatus.

Fourteen pure right-handed (H.N. handedness inventory [20], all participants ≧8, mean = 9.1, *SE* = 0.26) university students (aged 19–27 years of age, mean = 20.0; nine men, five women) participated in the experiment. All subjects normally operated a computer mouse with their right hand. The H.N. handedness inventory is a revised version of the Edinburgh Inventory [21] for Japanese subjects. Participants respond to this scale by indicating whether they use their right, left, or either hand for 10 common actions. This scale ranges from −10 to +10; a “right” response is scored as +1, a “left” response is scored as −1 and a response of “either” is scored as zero. We obtained written informed consent from all of the participants before conducting the experiment. All participants reported normal or corrected-to-normal vision, hearing, and somatosensation and no neurological abnormalities. We used MATLAB (MathWorks, Natick, MA, USA) and Psychophysics Toolbox [22], [23] to create the visual stimuli and conduct the experiment. The stimulus was a cursor on a virtual display screen (1024×768 pixels); the cursor moved in response to movement of a wireless mid-space mouse (Fig. 1). The display device (GVD-510-3D, Shenzhen Oriscape Electronic, Shenzhen, Guangdong, China) was attached to the chin rest and the participant was looking through the device that gives the image of a 28° visual angle virtual screen. Participants were not able to see their arms. The mid-space mouse (BOMU-W24A/BL, Buffalo, Nagoya, Japan) weighed 135 g and could be used in the air because of its gyroscopic sensor. We avoided the effect of motor training resulting from prior experience (e.g., typical right-handed mouse use) by ensuring that none of the participants had used our particular equipment before the experiment.

### Procedure

We asked participants to move the mouse to point the cursor in a straight line to the target, which was located somewhere on a circle 500 pixels in diameter on the display screen, and to click the mouse button when they finished moving the cursor. The participants were instructed to assume that the screen was located just in front of their reach and that the cursor position was synchronized with the mouse position. The cursor speed was set so that the cursor position on the 60 cm distant screen was similar to that of the mouse in the air (see Fig. 1).

Under the visual-feedback condition, the participants could see the cursor in response to the mouse movement. In contrast, under the no-visual-feedback condition, the cursor disappeared when it went beyond a circle 100 pixels in diameter centered on the starting point. Thus, participants had to click on the target without visual feedback by predicting their arm movement. They did not know the task condition until they actually moved the mouse. Participants moved the mouse with right, left, or both hands. That is, we used six conditions (two visual feedback conditions × three hand conditions), and participants engaged in 30 trials (six conditions × five repetitions) arranged in random order.

In each trial, the participants first set the mouse at the supposed starting point (2×2 pixels at the center of the screen) with the indicated arm(s) straight. When they finished setting the mouse, they clicked the mouse button once. They were then instructed to try to alternate clicking the mouse with the right and the left hand under the both-hands condition. After a random interval (from 1 to 3 s), the cursor was set at the start point and the target (2×2 pixels) appeared. Participants then moved the mouse device in a straight line to the target and clicked the mouse as quickly but accurately as possible. In the no-visual-feedback condition, after clicking the button to indicate the target, the cursor appeared again so that participants could view their accuracy. We recorded the final location of the cursor and the duration of time required to complete the task.

### Data analysis

We calculated four measures from these data. For movement error, we determined the Euclidean distance between the target and the point at which the participants clicked in each trial. For reaction time, we determined the length of time between the cursor and target appearances and when their cursor went beyond the 100 pixel diameter circle. We measured the time that had elapsed between the movement of the cursor beyond the 100-pixel-diameter circle and the final click on the target on the 500-pixel-diameter circle to determine the movement time. This duration was measured because micro-motions of the mouse occurred even at the starting point, making it difficult to determine when the first movement began. To measure target overshooting or undershooting, we transformed the Euclidean distance between the target and the clicked point so that negative values indicated undershoots and positive values indicated overshoots [14].

The protocol of the present study was approved by the local ethics committee (The Ethical Committee on Human Experimentation of the Graduate School of Arts and Sciences, The University of Tokyo).

## Results

**Figure 2:**
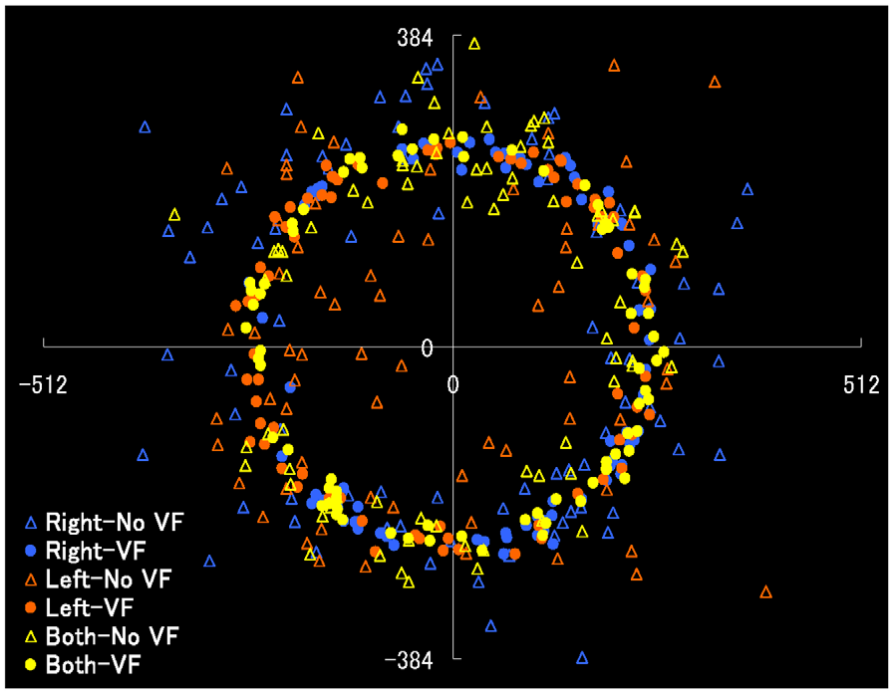
Relationship among clicked points, hand usage (left, right, both), and visual feedbacks (VF).

**Figure 3:**
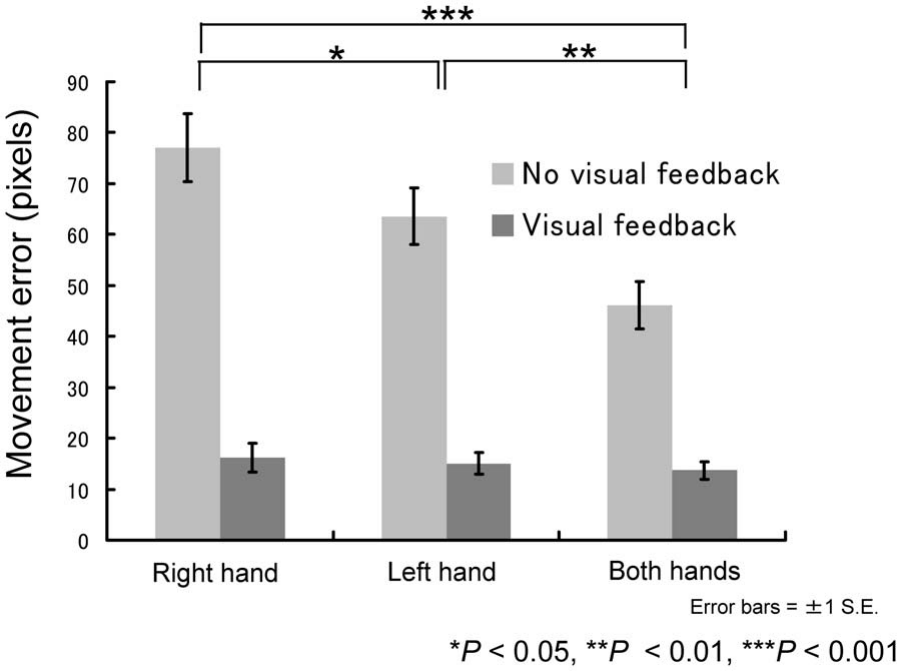
Relationship among movement error, hand usage, and visual feedback.

It was not surprising that although the clicked points under the visual-feedback condition were generally arranged in a circle, those under the no-visual-feedback condition were scattered (Figure 2). We first examined the effect of visual feedback and hand condition on the movement error (Figure 3). Analysis of movement errors with a two-way repeated-measures analysis of variance (ANOVA) revealed significant main effects for the factors of “feedback” (*F*1,13 = 221.1, *P*<0.01) and “hand condition” (*F*2,26 = 11.8, *P*<0.01) and for the interaction of “feedback x hand condition” (*F*2,26 = 7.26, *P*<0.01). The simple main effect of “feedback” was significant under each hand condition, and the simple main effect of “hand condition” was significant under the no-visual-feedback condition (*P*<0.001 in all cases). A *post-hoc* multiple comparison using Ryan's method (i.e., R-E-G-W's *F*-test) revealed significant differences between the right-hand and bimanual conditions, the left-hand and bimanual conditions, and the right- and left-hand conditions (*P*<0.05) under the no-visual feedback condition.

**Figure 4:**
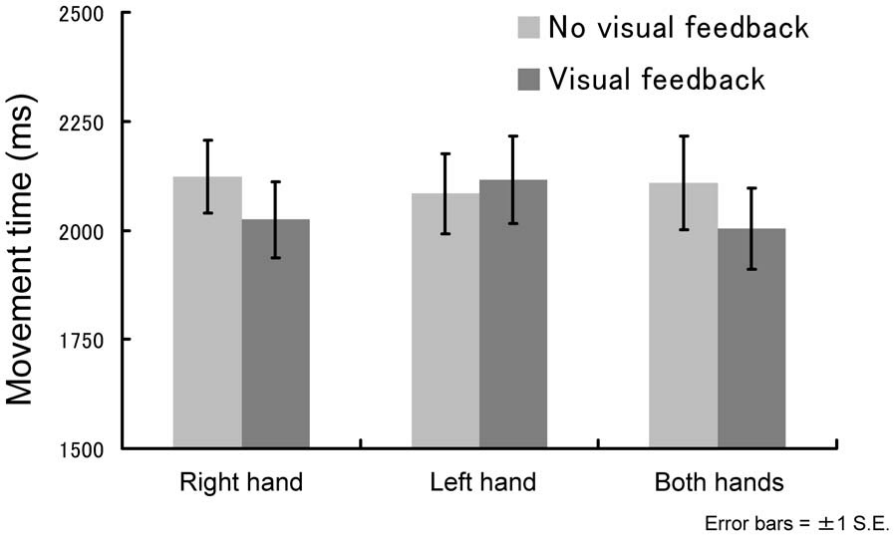
Relationship among movement time, hand usage, and visual feedback.

Figure 4 shows the relationships among movement time, hand condition, and visual feedback. However, analysis of movement times with a two-way ANOVA similar to that described above revealed that the main effects for “feedback” (*F*1,13 = 0.04, *P* = 0.85) and “hand condition” (*F*2,26 = 0.03, *P* = 0.97) and the interaction of “feedback x hand condition” (*F*2,26 = 0.67, *P* = 0.52) did not reach statistical significance.

**Figure 5:**
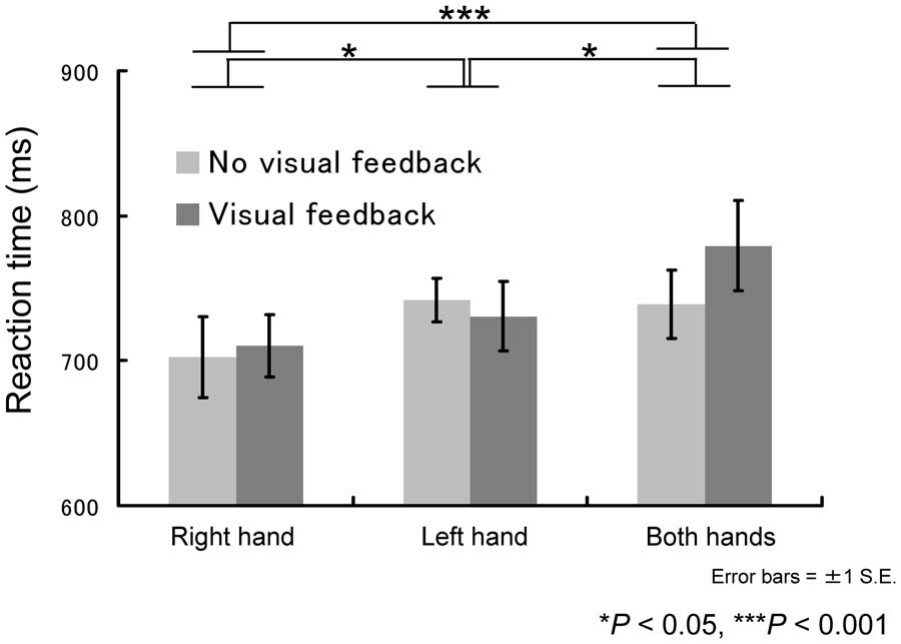
Relationship among reaction time, hand usage, and visual feedback.

Analysis of reaction times with a two-way ANOVA similar to that described above (Fig. 5) showed a significant main effect for only “hand condition” (*F*2,26 = 11.52, *P*<0.001); the main effect of “feedback” (F1,13 = 1.33, *P* = 0.27) and the interaction of “feedback x hand condition” (*F*2,26 = 1.13, *P* = 0.34) were not significant. A post-hoc multiple comparison using Ryan's method revealed significant differences between the right-hand and bimanual conditions, left-hand and bimanual conditions, and right- and left-hand conditions (P<0.05). It is reasonable that there was no difference between the visual-feedback and no-visual-feedback conditions because participants could not know which was in effect until the cursor went beyond the designated circle radius. The right hand, which had the shortest reaction time, had the largest movement error, and both hands, which had the longest reaction time, had the smallest movement error. This suggests that movement error under the no-visual-feedback condition is related to reaction time, rather than movement time.

To examine the relationship between movement error and reaction or movement time, we conducted Pearson's correlation analysis (collapsed across hand conditions for purposes of simplification). Under the no-visual feedback condition, the relationship between movement error and reaction time (*r* = −0.20, n.s.) and that between movement error and movement time (*r* = −0.28, n.s.) were not significant. In addition, neither the former (*r* = −0.26, n.s.) nor the latter (r = −0.27, n.s.) relationship was significant under the visual feedback condition. These results suggest that a simple trade-off relationship (e.g., between longer reaction time and less movement error) might not obtain.

**Figure 6:**
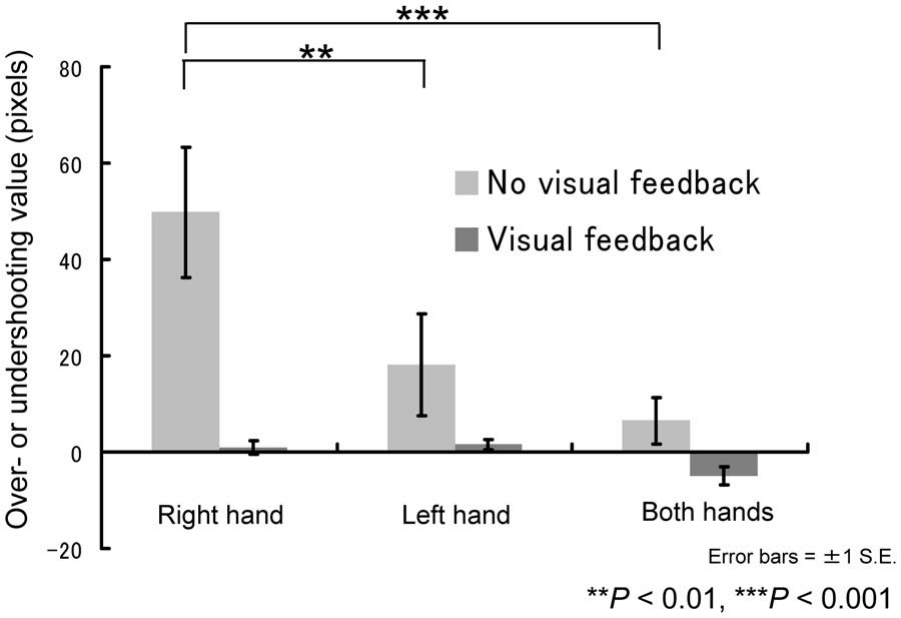
Relationship among overshooting, hand usage, and visual feedback. The negative values indicate undershoots and positive values indicate overshoots.

Finally, to examine the potential tendency of movement error, we focused on the relationship of overshooting, hand condition, and visual feedback (Figure 6). Analysis of overshooting with a two-way ANOVA revealed significant main effects for “feedback” (*F*1,13 = 11.7, *P*<0.01) and “hand condition” (F2,26 = 5.71, *P*<0.01) and for the interaction of “feedback x hand condition” (*F*2,26 = 4.18, *P*<0.05). The simple main effect of “feedback” was significant under the right-hand condition (*P*<0.001), and the simple main effect of “hand condition” was significant under the no-visual-feedback condition (P<0.001). A post-hoc multiple comparison using Ryan's method revealed significant differences between the right-hand and bimanual conditions and between the right- and left-hand conditions (P<0.01) for the no-visual-feedback condition. Thus, the right hand under the no-visual-feedback condition tended to overshoot the targets (see Figure 2).

## Discussion

The present study examined how each hand performs differently during reaching tasks with and without visual feedback as well as how bimanual reaching differs from unimanual reaching. Interestingly, when we focused on movement errors committed by our right-handed sample, left-hand movements were associated with smaller errors than right-hand movements under the no-visual feedback conditions, whereas no differences emerged under the visual feedback conditions. Furthermore, bimanual coordinated reaching was associated with smaller errors than was unimanual reaching. These results might reflect the special abilities of each hand with respect to motor control that are derived from internal models.

Internal models of motor control allow humans to move the body smoothly despite, for example, delays of several hundred milliseconds for signals to transfer between the brain and an arm [24]. This idea of internal models as representations within the central nervous system that simulate the naturally occurring transformations between sensory signals and motor commands has become a central theoretical framework to the understanding of the neural control of movement [25], [26]. Once a person develops an internal model, he or she can control the motor system in a feed-forward (predictive) way [27]. Most studies of internal-model learning have examined learning in the dominant hand [18], [19], [28]). However, coordinated bimanual actions form the basis for many everyday motor skills. Visually guided reaching and grasping may be the most basic actions of humans.

### Right-hand versus left-hand reaching

Some previous studies have suggested similar results in terms of the performance differences between the right and left hands [8], [14], [29]. One explanation is that the right-hemisphere advantage for the spatial planning of movements in right-handers produces pronounced accuracy advantages for the left hand [14]. Furthermore, the larger movement error in right-hand conditions might be related to overshooting the target. A previous study [30] and the present study found overshooting with the preferred hand, whereas another study reported undershooting with the preferred hand [14]. Since a space-coordinate mismatch between movement and vision was sometime inevitable in the present and the previous studies (discussed later), over- or undershooting might depend on the experimental setting. The relationship between predictive motor control error and overshooting (or undershooting) should be examined in the future.

When reaction times and movement error were considered, more interesting results appeared. Since we determined the reaction time as the length of time between the appearances of the cursor and target and when the cursor went beyond the 100-pixel-diameter circle, the reaction times that we observed might be longer than those reported in previous studies [5]. Although a previous study suggested that left-hand movement reaction time might be shorter than that for the right hand [31], we found that the right-hand reaction time was shorter. Reaction time is considered a measure of movement planning [32]. That is, the right hand may react more rapidly but have larger movement error, whereas the left hand may react more slowly but have smaller error. It is well established that the preferred hand outperforms the non-preferred hand with regard to the online control of the final corrective stage of aiming movements [5], [33], [34]. Therefore, the controller of the preferred hand might be more adapted for closed-loop movement control (feedback control), whereas the controller of the non-preferred hand seems to be better prepared for open-loop movement control (feed-forward control) [14]. Thus, for the left hand, more time is needed to compute motor planning more accurately, but once the computation is finished, the left hand is better controlled than the right, even without visual feedback. For the right hand, less motor-planning time is needed, but the computation may be less accurate; however, the right hand may be controlled online with visual feedback. As a result, under visual-feedback circumstances, the right hand performs better [5]or as well as the left hand [35].

The hypothesis that closed-loop control of the right hand and open-loop control of the left hand might be congruent with the aforementioned traditional notions regarding hemispheric dichotomization insofar as the left hemisphere (controlling the right hand) has appeared to be especially well-equipped to perform tasks related to sensory feedback, while the right hemisphere (controlling the left hand) has appeared to be especially well-equipped to perform tasks related to spatial planning. If, however, this hypothesis were accurate, the right hand would have outperformed the left hand in terms of the accuracy of the responses in the presence of visual feedback, and more time would have been required to perform movements when they were controlled by feedback, as was found in previous studies. In the presence of visual feedback, the advantage of the right hand with respect to accuracy and the consequently longer time devoted to movement may emerge only when greater precision is required to perform the experimental task [36]. In the present study, we emphasized both speed and accuracy to reflect the functional differences between the two internal models; this procedure may have resulted in the absence of an advantage for the right hand under the visual feedback condition because participants might not have consulted the visual feedback sufficiently when moving their hands. These results suggest that the two hands have different specialties of motor control for various types of cases, rather than one hand being superior to the other. Such specialties must be based on different internal models [35]. Lenhard and Hoffman [14]claimed that this functional difference could, for example, be a direct result of more frequent supervision of the preferred hand as compared to the non-preferred hand.

### Unimanual versus bimanual reaching

Our comparison of unimanual and bimanual movements showed that bimanual movement error was smaller than unimanual movement in the no-visual-feedback condition. This finding cannot be explained by bimanual coordinative movement as a means of sharing weight or stabilizing the body. If bimanual stabilizing with the hands makes accurate motor control possible, bimanual movement time and movement error under a visual-feedback condition should have decreased. Rather, we suggest that the results are related to motor prediction or planning, which the internal model, especially the forward dynamic model [27], makes possible. Bimanual internal model coordination may produce more accurate motor prediction. Sainburg and Kalakanis may suggest that the control of the two limbs is mediated by distinct neural mechanisms and different internal models [35]. Lenhard and Hoffman reported that whereas variable aiming error tended to be lower for the preferred hand, constant aiming error was consistently lower for the non-preferred hand [14]. These findings support the idea of a spatial accuracy advantage for the controller of the non-preferred hand and the suggestion that the two hands have different internal models and motor planning processes. It is reasonable to suggest that two different internal models could compute and predict our movements more accurately than one internal model, because accuracy would improve with multiple modeling (e.g., multiple models for motor control [37]). If reaction time can be considered a measure of movement planning [32], the result that bimanual reaction time was longer than unimanual reaction time means that bimanual motor planning took longer. It is possible but reasonable that computation using two internal models takes more time but yields more accurate motor prediction or planning compared to the use of a single unimanual internal model.

### An alternative interpretation and the limitations of the present study

In this context, alternative interpretations must be considered. Results showing that movement prediction errors increased as the preparation times for such movements decreased may appear self-evident (speed-accuracy trade off). The Pearson correlation analysis between movement prediction error and movement preparation time revealed no significant association. That is, these findings were not consistent with the possibility that longer preparation was associated with better prediction, regardless of the particular hand used. Even if this is true temporally (a negative r value might indicate this possibility, although it was not statistically significant), it is unclear why this speed/accuracy trade-off pattern appeared among the hand conditions used in this study. All else being equal, these discrepancies might indicate differences in the internal models; that is, the right hand emphasizes speed and the left hand emphasizes accuracy, and these differential emphases would be learned, practiced, and adjusted to the situation according to the innate functional differences in the cerebral hemispheres.

Although this study yielded interesting results, the findings are limited by the constraints inherent in the experimental situation. Previous studies have used a mouse (or pointing) device and the display to control visual feedback [14], [38]. However, these circumstances make it difficult to match movement with visual spatial coordinates, because the display must be placed in front of the arms [14], [39] or at a location that obscures [38] the arms from view. These conditions inevitably result in a space-coordinate mismatch between movement and vision. In the present study, we proposed a new method using a mid-space mouse and a virtual display that enabled the matching of these coordinates so that arm movements did not prevent visual feedback via a virtual screen. Nonetheless, even this experimental setting could not provide perfect matches for these space coordinates. Future studies must still resolve the lingering methodological issues.
